# Pioneering Study on *Rhopalurus crassicauda* Scorpion Venom: Isolation and Characterization of the Major Toxin and Hyaluronidase

**DOI:** 10.3389/fimmu.2020.02011

**Published:** 2020-08-20

**Authors:** Caio B. Abreu, Karla C. F. Bordon, Felipe A. Cerni, Isadora S. Oliveira, Carla Balenzuela, Gabriel M. Alexandre-Silva, Karina F. Zoccal, Mouzarllem B. Reis, Gisele A. Wiezel, Steve Peigneur, Ernesto L. Pinheiro-Júnior, Jan Tytgat, Tiago M. Cunha, Loic Quinton, Lúcia H. Faccioli, Eliane C. Arantes, Umberto Zottich, Manuela B. Pucca

**Affiliations:** ^1^Medical School, Federal University of Roraima, Boa Vista, Brazil; ^2^Department of BioMolecular Sciences, School of Pharmaceutical Sciences of Ribeirão Preto, University of São Paulo, São Paulo, Brazil; ^3^Barão de Mauá University Center, Ribeirão Preto, Brazil; ^4^Department of Clinical Analysis, Toxicology and Food Science, School of Pharmaceutical Sciences of Ribeirão Preto, University of São Paulo, São Paulo, Brazil; ^5^Toxicology and Pharmacology, KU Leuven, Leuven, Belgium; ^6^Department of Pharmacology, Ribeirão Preto Medical School, University of São Paulo, São Paulo, Brazil; ^7^Mass Spectrometry Laboratory, MolSys Research Unit, Liège Université, Liège, Belgium

**Keywords:** scorpion venom, *Rhopalurus crassicauda*, toxin, electrophysiology, nociception, neurotoxin, pro-inflammatory toxin

## Abstract

Scorpionism is responsible for most accidents involving venomous animals in Brazil, which leads to severe symptoms that can evolve to death. Scorpion venoms consist of complexes cocktails, including peptides, proteins, and non-protein compounds, making separation and purification procedures extremely difficult and time-consuming. Scorpion toxins target different biological systems and can be used in basic science, for clinical, and biotechnological applications. This study is the first to explore the venom content of the unexplored scorpion species *Rhopalurus crassicauda*, which inhabits exclusively the northernmost state of Brazil, named Roraima, and southern region of Guyana. Here, we pioneer the fractionation of the *R. crassicauda* venom and isolated and characterized a novel scorpion beta-neurotoxin, designated Rc1, and a monomeric hyaluronidase. *R. crassicauda* venom and Rc1 (6,882 Da) demonstrated pro-inflammatory activities *in vitro* and a nociceptive response *in vivo*. Moreover, Rc1 toxin showed specificity for activating Na_v_1.4, Na_v_1.6, and BgNa_v_1 voltage-gated ion channels. This study also represents a new perspective for the treatment of envenomings in Roraima, since the Brazilian scorpion and arachnid antivenoms were not able to recognize *R. crassicauda* venom and its fractions (with exception of hyaluronidase). Our work provides useful insights for the first understanding of the painful sting and pro-inflammatory effects associated with *R. crassicauda* envenomings.

## Introduction

Venomous animals possess the capacity to develop a wide array of compounds with different biological effects inside a specialized apparatus to inject the venom into the preys (1, 2). In Brazil, accidents caused by venomous animals are a frequent neglected disease, with blind spots regarding general education and proper approach in most countries (3, 4). Scorpionism is included in this scenario, in which *Tityus serrulatus* species is responsible for most of the accidents in the country, reaching numbers of over 100,000 reports in 2017 (5–7). In spite hereof, there are still many neglected accidents caused by other scorpion species in the Brazilian biome (6), such as the scorpion *Rhopalurus crassicauda*, which make the data underestimated.

Described in 1947 (8), *R. crassicauda* species inhabits exclusively the northernmost state of Brazil named Roraima and southern region of Guyana (3), although species of the same genus are found in other regions of the country (9, 10). However, *R. crassicauda* taxonomic is complex and controversial. For instance, the enigmatic species was also referred as *R. pintoi* in spite of the deep differences between both original descriptions. Further, it was treated as a subspecies of *R. laticauda*, which was restored as *R. crassicauda* as the valid species (11). Recently, the species was again considered a *R. laticauda* species (12). In this study we will keep the original taxonomic classification endorsed by Lourenço (2002) – R. *crassicauda* (11), since the researcher performed *R. crassicau*da collections around Boa Vista, Roraima, where actual samplings for the present work were conducted.

Albeit underreported accidents are a problem throughout Brazil, Roraima stands out for its scarce investment in research, the high number of indigenous people (more than 40% of the state is considered indigenous areas), and the vast number of Venezuelan migrants (3). Moreover, Roraima is a very poor region and has been overlooked by the Brazilian government and richest states (i.e., located mainly in south and southeast regions). Although the envenomings caused by scorpions in Roraima have been increasing in wide scale (32, 139, and 288 cases in 2007, 2017, and 2019, respectively) (13), human envenomings caused by *R. crassicauda* and its venom composition remain unexplored (unmatched data in the main academic databases), even though more than 70 years passed since the species description.

Scorpion venoms can trigger several clinical effects and their toxins can target different biological systems (5). Thus, several signs and symptoms can be observed in victims stung by scorpions such as pain, myosis, bradycardia, cardiac arrhythmias, arterial hypotension, increased lachrymal, nasal, salivary, pancreatic, gastric and bronchial secretions, diaphoresis, tremors, piloerection, and muscle spasms, increases blood amylase levels, mydriasis, cardiac arrhythmias, tachycardia, arterial hypertension, acute pulmonary edema, cardiac failure, and even circulatory shock following death (14–16). Concerning *R. crassicauda*, although there is no report about the signs and symptoms, physicians in Roraima have not faced severe intoxication by victims stung by this species, although intense pain and mild paresthesia are always reported by patients.

In Brazil, the use of specific antivenom is indicated to treat all severe scorpion envenomings (5, 17), and the antivenom administration is mandatory in case of envenoming in children under 7 years-old or adults, mainly elderly, with previous health problems such as hypertension and cardiovascular diseases. Nevertheless, besides all the potential adverse effects produced by horse-derived antivenoms (i.e., anaphylactic reaction and serum sickness), and the larger than necessary dose of equine antibodies (about 70% of the antibodies are considered unspecific) (18), the Brazilian scorpion antivenom is produced exclusively against *T. serrulatus* species and could not be used to treat accidents caused by other scorpion genera (e.g., *R. crassicauda*), whilst there are more than 160 different documented scorpion species in the country (7). This study is the first one to explore the venom content of the neglected scorpion species *R. crassicauda* from an overlooked and poor state of Brazil – Roraima. In particular, an effort was made to isolate and characterize a novel scorpion neurotoxin (Rc1), and a spreading factor (hyaluronidase) from its venom.

## Results

### Scorpions and Venom Milking

**FIGURE 1:**
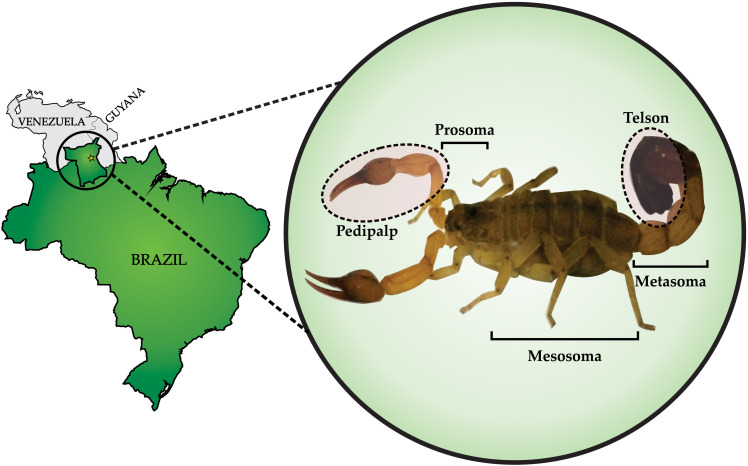
*Rhopalurus crassicauda*: collection and morphology. Left panel shows Roraima, the northernmost state of Brazil. The yellow star indicates the capital city of the state, Boa Vista, where *R. crassicauda* scorpions were collected. Zoom view in the right panel shows the *R. crassicauda* species (scorpion length – 10 cm). Photo taken of a specimen kept in the scorpion *vivarium* of the research group.

A total of 23 specimens of *R. crassicauda* scorpions, collected in the region of Boa Vista (Figure 1), were kept in the *vivarium* for venom milking. In order to standardize venom milking, our research group successfully built a restraining electrical device coupled to a dimmer potentiometer. After different electrical stimulation tests in the scorpion telson, the voltage of 18 V proved to be the best and thus suitable for *R. crassicauda* milking. During the initial stages of electrical stimulation, a colorless, watery venom was obtained; subsequently, the milked venom was a viscous fluid. From a total of 115 milky scorpions (one milky droplet extracted), 9.2 mg of proteins was estimated in the soluble crude venom, corresponding to an average of 80 μg of proteins per scorpion.

### Venom Fractionation and Enzymatic Activities

**FIGURE 2:**
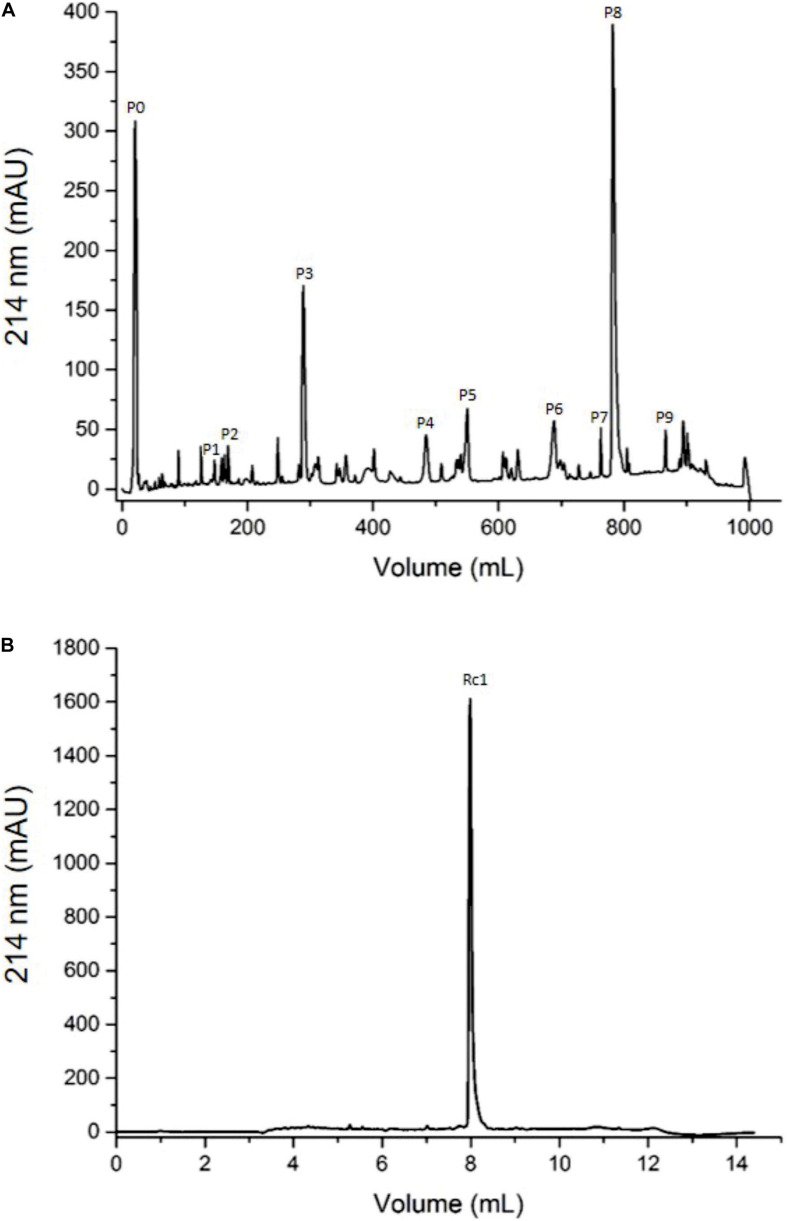
Chromatographic profiles of *R. crassicauda* venom using RP-FPLC system. The protein elution was carried out in a segmented concentration gradient from 0 to 100% of solution B (80% ACN in 0.1% TFA) and absorbance was monitored at 214 nm. **(A)** *R. crassicauda* venom (2 mg) was eluted using 5 concentration gradient steps on a C18 column (250 × 10 mm, 300 Å, and 5 μm particles), at a flow rate of 5 mL/min. **(B)** P8 (40 μg) was re-chromatographed using 4 concentration gradient steps on a C18 column (250 × 2.1 mm, 300 Å, and 5 μm particles), at a flow rate of 0.5 mL/min.

To isolate the toxins, the *R. crassicauda* venom was submitted to reversed-phase fast protein liquid chromatography (RP-FPLC) on a C18 column (10 × 250 mm) and the major peak (fraction P8, Figure 2A) was re-chromatographed on a different C18 column (2.1 × 250 mm; Figure 2B). The resulting pure toxin, named Rc1, represented 24% of the total protein of the soluble crude venom.

**FIGURE 3:**
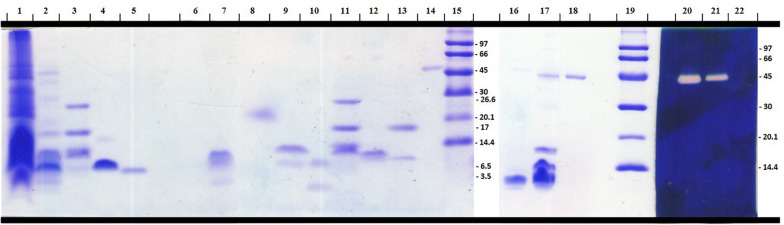
Electrophoretical profile of *R. crassicauda* venom and fractions. Tris-tricine-SDS-PAGE (16.5%) under reducing (Lanes 1–15) and non-reducing (Lanes 16–22) conditions. Lanes 16–22 were incorporated with hyaluronan before gel polymerization. Lanes 1, 16, and 22: *T. serrulatus* venom (18, 16, and 5 μg, respectively). Lanes 2, 17, and 21: *R. crassicauda* venom (18, 5, and 5 μg, respectively). Lanes 3 and 11: molecular mass markers (Sigma M3546). Lanes 15 and 19: molecular mass markers (GE Healthcare 17-0446-01). Lanes 4 and 5: Ts1 and Rc1 (P8), respectively (2 μg). Lanes: 6: P1; 7: P2; 8: P3; 9: P4; 10: P5; 12: P6; 13: P7 (all 2 μg). Lanes 14, 18, and 20: P9 (2 μg). Lanes 1–19 were stained with PlusOne Coomassie Blue PhastGel^®^ R-350. Lanes 20–22 were stained with Stains-all for evaluation of the hyaluronidase activity.

A Tricine-SDS-PAGE (16.5%) electrophoresis was used to evaluate the complexity of the components present in *R. crassicauda* venom compared to *T. serrulatus* venom, as well as the purity profile of the eluted fractions (Figure 3). Non-reduced venom and peak 9 (P9) showed a single translucent band of ∼45 kDa in the hyaluronan-based gel (Figure 3, lanes 20 and 21), indicanting hyaluronidase activity. On the other hand, under reduction conditions, P9 presented a molecular mass of ∼54 kDa (Figure 3, lane 14). The major peak P8 revealed a unique protein band of ∼6.5 kDa (Figure 3, lane 5).

Non-enzymatic activities of PDE and PLA_2_ were detected on *R. crassicauda* venom in the tested concentrations (5 and 65 μg/well, respectively, data not shown).

### Molecular Mass and Sequence of Rc1

**FIGURE 4:**
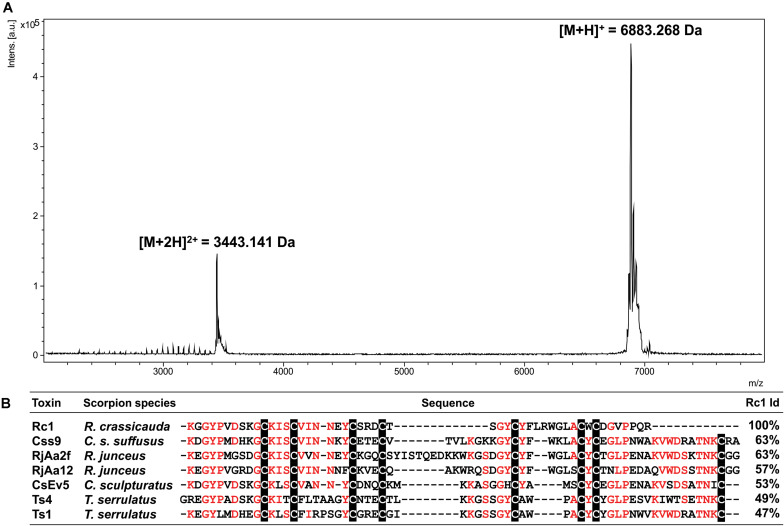
**(A)** Mass spectrum of Rc1 obtained by MALDI-TOF mass spectrometry in a positive linear ionization mode using DHB matrix. **(B)** Sequence alignments of the Rc1 partial sequence (B3EWP2) with other beta-neurotoxins. Css9 (*Centruroides suffusus*, F1CGT6), CsEv5 (*C. sculpturatus*, P58779), Ts4 (*T. serrulatus*, P45669), Ts1 (*T. serrulatus*, P15226), RjAa2f (*R. junceus*, E7CLP6), and RjAa12 (*R. junceus*, E7CLN6). Conserved residues are in red and Cys residues are shaded in black. Alignment was generated by Clustal Omega server. Identity percentage considered among the aligned residues.

The mass spectrum of Rc1 showed an average ion *m/z* 6,883.3 Da and the ion *m/z* 3,443.1 Da [(M + 2H)^2+^; Figure 4A]. The first 27 N-terminal amino acids from Rc1 were determined by Edman degradation method as KGGYPVDSKGCKISCVINNEYCSRDCT. In addition, Rc1 internal peptides (Supplementary Table 1) were determined by *de novo* sequencing resulting in a sequence coverage of ∼80%, considering the peptides masses in comparison to Rc1 molecular mass determined by matrix-assisted laser desorption/ionization (MALDI)-time of flight (TOF; Figure 4A). Seven Cys conserved residues were recovered from 8 estimated Cys residues. The total sequence obtained for Rc1 (the protein sequence data will appear in UniProt Knowledgebase under the accession number C0HLR6) presented 60% identity with Css9 beta-neurotoxin from *Centruroides suffusus* (Figure 4B).

### Antivenom Cross-Reactivity

**FIGURE 5:**
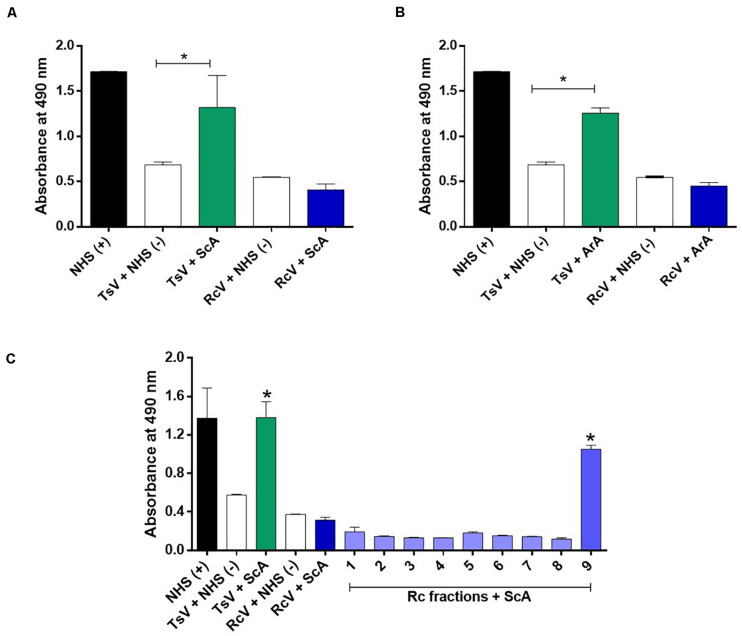
Immunorecognition of *R. crassicauda* venom by Brazilian scorpion and arachnid antivenoms. **(A)** Scorpion antivenom. **(B)** Arachnid antivenom. **(C)** Scorpion antivenom and *R. crassicauda* fractions. The 96-well plates were coated with 2 μg of *R. crassicauda* venom or fractions (1–9) diluted in a solution of 0.05 M carbonate-bicarbonate buffer (pH 9.6). Positive controls (+) were performed with wells coated with non-immune horse serum or TsV, and negative controls (−) were performed by replacing antivenoms with non-immune horse serum. Absorbance was measured at 490 nm. NHS: non-immune horse serum. TsV, *T. serrulatus* venom; ScA, scorpion antivenom; ArA, arachnid antivenom; and RcV, *R. crassicauda* venom. Results are presented as mean ± SD (*n* = 3), which were analyzed by ANOVA followed by Tukey’s multiple comparison test (*p < 0.05, when compared to the negative respective controls).

The Brazilian scorpion (ScA) and arachnid (ArA) antivenoms were not able to recognize the soluble crude venom of *R. crassicauda* (Figures 5A,B). However, ScA was able to recognize venom fraction P9, which presents hyaluronidase (Figure 5C).

### Venom and Major Toxin Effects

Both *R. crassicauda* venom and Rc1 toxin (100 and 50 μg/mL, respectively) demonstrated no cytotoxic effects on J774.1 cells (data not shown).

**FIGURE 6:**
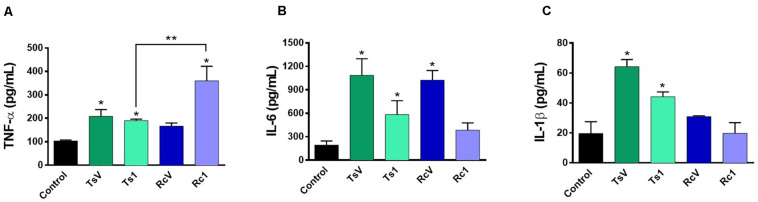
Effect of *R. crassicauda* venom and the major toxin on cytokine levels. J774.1 cells were stimulated with *R. crassicauda* venom (100 μg/mL) or Rc1 toxin (50 μg/mL) for 24 h. As negative control, stimuli with *T. serrulatus* venom (100 μg/mL), Ts1 (50 μg/mL), and unstimulated cells were used. **(A)** TNF-α. **(B)** IL-6. **(C)** IL-1β. TsV: *T. serrulatus* venom. RcV: *R. crassicauda* venom. Results are presented as mean ± SD (*n* = 4), which were analyzed by ANOVA followed by Tukey’s *post hoc* test (*p < 0.05 when compared to controls; **p < 0.001 when compared to Ts1).

**FIGURE 7:**
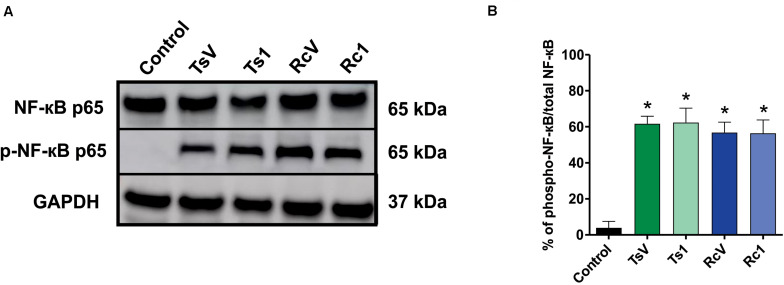
Effect of *R. crassicauda* venom and the major toxin on the NF-kB signaling pathway. **(A)** Western blot of phospho-NF-kB p65 and NF-kB p65 proteins (GAPDH as an internal control). Lane 1 represents control (medium) and lanes 2–5 represent TsV, Ts1, RcV, and Rc1, respectively. **(B)** Percentage of expression of the target protein against the reference protein as quantified by band densitometry. TsV: *T. serrulatus* venom. RcV: *R. crassicauda* venom. Results are presented as mean ± SD (*n* = 4), which were analyzed by ANOVA followed by Tukey’s *post hoc* test (*p < 0.001 when compared to control).

*Rhopalurus crassicauda* venom (100 μg/mL) increased IL-6 production (Figure 6B), whereas Rc1 stimulated TNF-α production. Interestingly, Rc1 was able to increase two-fold the TNF-α levels when compared to Ts1 (Figure 6A), being a potent inflammatory toxin. Moreover, NF-kB signaling pathway was activated by *R. crassicauda* venom and Rc1 stimuli, as observed with *T. serrula*tus venom and Ts1, indicating that both scorpion venoms, and major toxins activate intracellular pro-inflammatory pathways (Figure 7).

**FIGURE 8:**
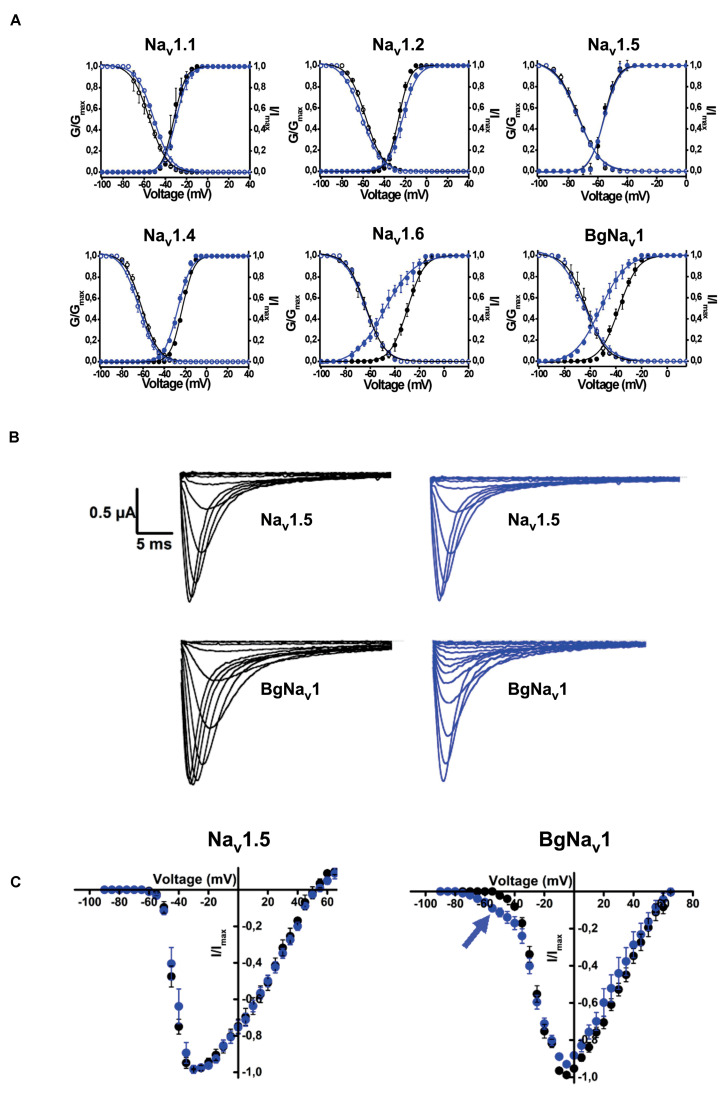
Electrophysiological effects of the major toxin on voltage-gated sodium channels. **(A)** Effects of Rc1 on the voltage dependence of steady-state activation and inactivation curves under control conditions (black symbols) and after the addition of 1 μM of Rc1 (blue symbols), *n* = 4 cells ± SEM. **(B)** Current traces of Nav1.5 (non-effect) and BgNav channels in control (black) and in the presence of Rc1 channels (blue). **(C)** IV curves in control (black) and after application of Rc1 for Nav1.5 (non-effect) and BgNav1 channels.

Rc1 activity was also tested on 6 different voltage-gated sodium channels expressed in *Xenopus laevis* oocytes. It was investigated if Rc1, at a concentration of 1 μM, could modulate the voltage dependence of the steady-state activation and inactivation curves. Rc1 altered the activation process of Nav1.4, Nav1.6 channels, and of the insect channels BgNa_v_1 (Figure 8). Application of 1 μM Rc1 shifted the midpoint of activation from −29.7 ± 0.1 mV in control to −48.5 ± 0.5 mV for Nav1.6 channels (*n* = 4). For BgNav1 channels, an alteration of the V_1/2_ from −37.7 ± 0.3 mV to −64.8 ± 0.5 mV after application of Rc1 was observed (*n* = 6). For Nav1.4 channels, a minor but still significant modulation of activation was noted since V_1/2_ values yielded −22.9 ± 0.1 mV and −27.2 ± 0.2 mV in control and in presence of Rc1, respectively. The steady-state inactivation curves were not significantly altered in the presence of Rc1. Rc1 did not show activity on Nav1.1, Nav1.2, and Nav1.5 channels (Figure 8).

**FIGURE 9:**
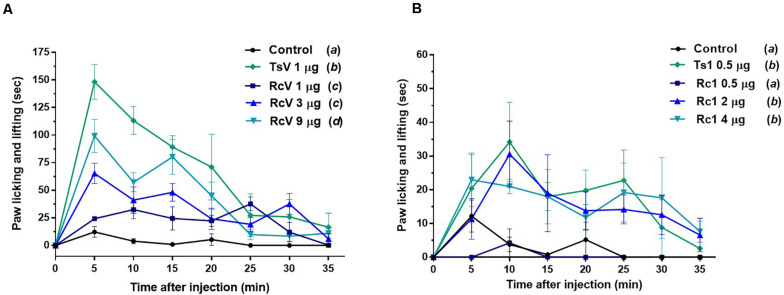
Spontaneous nociception induced by *R. crassicauda* venom and the major toxin. Nociception was assessed by recording the time course of paw licking and lifting behavior after intraplanar (ipl) injections of RcV venom **(A)** or Rc1 **(B)** into C57BL/6 mice right hind paw. *T. serrulatus* venom and its main toxin (Ts1) were used as controls. TsV: *T. serrulatus* venom. RcV: *R. crassicauda* venom. Data are presented as the mean ± SD (*n* = 5), which were analyzed by Two-way ANOVA followed by Tukey’s *post hoc* test. Same and different letters represent, respectively, no and statistically significant differences between groups.

*In vivo*, although *R. crassicauda* induced a significant increase in mice paw licking and lifting during the first 15 min (in all tested concentrations), the spontaneous nociception behavior was considerably lower when compared to *T. serrulatus* venom (Figure 9A). The same was observed to Rc1 toxin, which needs four-fold more toxin to induce similar nociceptive behavior of Ts1 (Figure 9B).

## Discussion

A single venom can contain up to several hundred different components producing diverse pathophysiological effects (19). Thus, studies focusing on the development of new drugs based on novel toxins remain valuable today. In addition, the understanding of a venom content can also elucidate local human envenomings and improve the efficacy of antivenom manufacture (20, 21). Although uncountable venomous species and their venoms have been over studied so far, *R. crassicauda* venom remains still unexplored (11).

In this study, the electrical method of venom extraction appears to be safe and successful for specimens of *R. crassicauda*, i.e., permanent injuries were not noticed neither immediately nor within 3 weeks after the milking. The 18 V required for *R. crassicauda* milking demonstrated to be higher than that used in the literature for *T. serrulatus* venom extraction (12 V) (22). The thickening of *R. crassicauda* metasoma probably explains the need for a higher tension (the prefix “crassi” means tick or fat in Latin, hence crassicauda denotes fat-tail).

The purification procedure of *R. crassicauda* venom was able to provide its pure major toxin Rc1 (P8) and hyaluronidase (P9). MS/MS analysis and N-terminal sequencing enabled to cover ∼80% of Rc1 primary sequence. Gaps are due to possible basic regions in Rc1 over digested by trypsin hindering peptide identification. It is interesting to note the presence of small fragments from the same peptide (Supplementary Table 1) which indicates that Rc1 may undergo a proteolytic cleavage by peptidases within the venom gland. In fact, peptidases have been already detected in scorpion venom glands such as those from *Mesobuthus eupeus* (ENA| EF442061.1) and *Hadrurus gertschi* (23). This process might be related to the processing of intracellular proteins as well as to increase venom complexity by producing different proteins/peptides from one single gene (23–26). A mechanism of post-splitting (a post-translational processing) was suggested to Ts19 from *T. serrulatus*, resulting on fragments with split functional activity (26). Moreover, toxin proteolysis is not uncommon to occur in scorpion venoms (24) and a similar fact have also been described in the venom from the ant *Neoponera villosa* (27).

Unfortunately, we could not explore the hyaluronidase sequence due to the low quantity of pure enzyme recovery. Hyaluronidases facilitate the spreading of toxins into the tissues of the prey/victims, since these enzymes hydrolyze hyaluronan of the interstitial matrix (28, 29). P9 is a monomeric hyaluronidase of 54 kDa (reduced) and 45 kDa (non-reduced), as estimated by Tris-Tricine-SDS-PAGE. The observed molecular mass is within the range from 45 to 82 kDa described for scorpion venom hyaluronidases (30, 31). Although there are about 2,200 scorpion species known in the world (32), there are only 12 scorpion hyaluronidases primary sequences deposited in the databanks and 5 enzyme isolation reports (from *Heterometrus fulvipes*, *T. serrulatus*, *Palamneus gravimanus*, *T. stigmurus*, and *Mesobuthus martensii* venoms) (28). Our study is the first one to isolate a hyaluronidase from the scorpion genus *Rhopaluru*s, as evidenced as a single band by Tris-Tricine-SDS-PAGE containing hyaluronan.

On the other hand, our study did not detect the phosphodiesterase (PDE) activity in Rc venom. PDEs are exonucleases, possessing the ability to cleave DNA and RNA, as well as other fundamental molecules for physiological processes such as ATP and cAMP (33, 34). These proteins have a molecular mass between 90 and 160 kDa and are widely distributed in snake venoms, although they are commonly found in a few amount on them (35, 36). So far, there are no reports describing PDEs in scorpion venoms. Indeed, the electrophoretic profile of Rc venom did not reveal proteins with high molecular mass over 54 kDa, which corresponds to hyaluronidase.

As such *Rhopalurus junceus* (37), *R. crassicauda* venom showed no PLA_2_ activity. Instead Rodríguez-Ravelo et al., using the same species (*R. junceus*) and mass spectrometry analysis, demonstrated that the scorpions from La Poa area, collected in the humid area of Baracoa, Guantanamo Province, showed the presence of phospholipase A_2_ (molecular mass within the range from 14 to 19 kDa) (38). The other scorpion species in which this enzyme activity was detected are *Pandinus imperator* (39, 40), *Anuroctonus phaiodactylus* (41), *Scorpio maurus palmatus* (42), *Opisthacanthus cayaporum* (43), *H. fulvipes* (44), and *H. laoticus* (45).

Rc1 exhibited an identity score in the range of 46–60% with scorpion β-neurotoxins from *C. suffusus, Centruroides sculpturatus*, and *T. serrulatus.*β-scorpion toxins bind at a so-called “site-4” of Na_V_ channels, and shift the voltage dependence of Na_V_ channel activation toward more negative potentials, promoting spontaneous and repetitive firing (46). Scorpion toxins targeting voltage-gated sodium channels (NaTx) are, in general, composed of 60–76 amino acids cross-linked by four disulfide bounds and are known as long-chain toxins (46). Rc1 presents the positively charged Lys at positions 1 and 12, but, strikingly, it does not show a negatively charged Glu at position 2. All these three residues were shown as determinants of the specificity of β-toxins (47, 48). In any case, the Rc1 electrophysiological findings for the first time reveal the neurotoxic effects of the main Roraima’s scorpion venom, which can explain some of the symptoms observed after local envenomings, such as local inflammation and pain.

Indeed, this is the pioneer study showing that *R. crassicauda* venom presents pro-inflammatory activities. The *in vitro* assays demonstrated that this species venom increases levels of IL-6 and that the main toxin Rc1 outstanding increases TNF-α levels. Both IL-6 and TNF-α are cytokines featuring pleiotropic activities. For instance, they can induce synthesis of acute phase proteins, stimulate antibody production and effector T-cell development, as well as elevation of body temperature (49, 50). On the other hand, in the tested concentrations, neither RcV nor Rc1 increased IL-1β, although high levels of this pro-inflammatory cytokine had previous been documented both in patients envenomated by *T. serrulatus* venom (51) and *in vivo* using *T. serrulatus* Ts6 toxin (52). In addition, our study demonstrated that both *R. crassicauda* venom and Rc1 can also activate the NF-kB signaling pathway. This result was consistent with previously reported results for *T. serrulatus* venom and toxins (5, 53–57), thus we supposed that activation of the NF-kB signaling pathway could be one important mechanism of enhancing the immune responses from several scorpion envenomings (53).

The NF-kB signaling pathway that mediates inflammatory responses is the canonical pathway, which is well described elsewhere (58, 59). Scorpion toxins, such as Ts1 (a β-toxin like Rc1), are known to be recognized by *toll like* receptors 2 (TLR2), 4 (TLR4), and CD14, resulting in the activation of NF-kB canonical pathway, which culminates in the production of inflammatory mediators such as cytokines (e.g., IL-6 and TNF-α) and lipid mediators (e.g., PGE_2_ and LTB_4_) (54).

Moreover, both RcV and Rc1 were not cytotoxic to macrophages in the tested concentration (100 μg/mL). Díaz-García et al. had demonstrated that different fractions of *R. junceus* venom were cytotoxic to A549 and MRC-5 lung cell lines; however, in that study, the authors tested high venom concentrations up to 600 μg/mL (37). In 2019, the same research group demonstrated that *R. junceus* venom inhibited the tumor progression in F3II bearing-mice in a dose-dependent manner (60). As such, *R. princeps* venom has also been explored as an anticancer agent (61).

Based on the greater number of neurotoxins affecting Nav channels and the increase of pro-inflammatory mediators, scorpion venoms can modulate the nociceptive response (5, 62). Factually, scorpions are well-known to cause immediate and localized painful stings, which can be classified as mild, moderate, severe, and very severe, being severe defined as a pain greater than that of a bee sting or equivalent (63, 64). Our studies demonstrated that both *R. crassicauda* venom as well as the toxin Rc1 are able to induce hypernociceptive response in mice, although to a lesser extend when compared to *T. serrulatus* venom and toxin. The peripheral sodium channels Na_v_1.3, Na_v_1.6, Na_v_1.7, Na_v_1.8, and Na_v_1.9 are mainly responsible for the pathophysiology of different pain syndromes (65, 66). Indeed, our electrophysiological studies conducted with isolated Rc1 toxin reveals that Rc1 alters the activation process of Nav1.4, Nav1.6, and BgNav1. Thus, the Rc1 action to Nav1.6 channels and the toxin-induced production of inflammatory mediators could explain the painful sting triggered by *R. crassicauda* envenoming. Finally, Rc1 also activated the insect channel BgNav from the cockroach *Blattella germanica*. Effects on insect ion channels are usually observed by scorpion-derived toxins, since insects are the preys of these animals, specially cockroaches, which are known to be their preferred diet (67). In fact, several studies demonstrate the potential of scorpion toxins to be used as insecticides (16, 68). Therefore, Rc1 can be classified as a β-scorpion toxin targeting mammal and insect voltage-gated sodium channels, pro-inflammatory, and painful neurotoxin. There are few studies developed with other *Rhopalurus* species that corroborate with our results. For instance, García-Gómez et al. demonstrated that the venom of *R. junceus* produces a β-effect on sodium channels in F11 cell line (69). Nonetheless, different from our work, many of the literature studies explore scorpion toxins targeting potassium channels (Kvs) (70).

Regarding the cross-reactivity observed on ELISA assays, our results suggest that none of the antivenoms evaluated can recognize *R. crassicauda* venom, indicating selectivity of both antivenoms toward *Tityus* spp. venom components. According to the Butantan Institute pipeline, ScA is an antivenom specific to *T. serrulatus* scorpion venom, being indicated to the treatment of envenomings caused by scorpion from *Tityus* genus; while ArA is a polyvalent antivenom produced against *T. serrulatus* and two different spider venoms (i.e., *Loxosceles* and *Phoneutria* genera), being indicated for the treatment of envenomings caused by scorpions and spiders. Knowing that *T. serrulatus* scorpions are responsible for most and severe cases of envenomings in Brazil, the ability of the available antivenoms to cross-neutralize venoms from others scorpion species is unknown, especially for accidents caused by another scorpion genus. In this study we did not identify antivenom cross-reactivity with *R. crassicauda* venom, although neurotoxins have been known to present a high degree of similarity (46, 71, 72). However, when we analyzed each venom fraction, the scorpion antivenom was able to recognize fraction 9 (P9), which corresponds to hyaluronidase. Since this enzyme is also found in *T. serrulatus* venom (73) and the ScA is produced against this species venom, it is not surprising that the antivenom can cross-bind to the hyaluronidase from *R. crassicauda* venom. Moreover, most of venom-derived hyaluronidases have demonstrated high sequence identities, specially between the same animal class (28). For instance, *T. serrulatus* hyaluronidase shares a high identity with hyaluronidases from the venoms of *C. sculpturatus* (XP_023226974.1, 76%), *M. martensii* (P86100.2, 72%), and *C. sculpturatus* (XP_023244120.1, 54%). The recognition of just one protein is definitely not enough to inhibit a venom cocktail toxicity and, although additional assays needs to be explored (i.e., *in vivo* lethality inhibition), it is unlikely that the scorpion antivenoms available in Brazil (ScA and ArA) could be used to treat severe cases of *R. crassicauda* envenomings in Roraima.

In conclusion, the present study pioneered the fractionation of *R. crassicauda* venom and successfully isolated and elucidated the major toxin, Rc1, and a hyaluronidase. Furthermore, this work provides useful insights for the first understanding of the painful sting and pro-inflammatory effects associated with *R. crassicauda* envenomings.

## Materials and Methods

### Scorpions and Venom Milking

*Rhopalurus crassicauda* scorpions were collected in Boa Vista city (latitude 2°49′14.88″ North and longitude 60°40′19.20″ West), Roraima (the northernmost state of Brazil; Figure 1). The scorpions were usually caught in the wild, and adults ranging in size from 3 to 5 inches (7–12 cm) were kept in plastic boxes with adequate ventilation. The identification of species were performed through the taxonomic key previously described (11). The animals received water daily, were fed with crickets or cockroaches at least twice a month, and were kept at Medical School of Federal University of Roraima with authorization from the Brazilian Biodiversity Information and Authorization System (SISBIO, http://www.icmbio.gov.br/sisbio/) number 57491.

In total, 23 scorpions were fed 5 days prior to venom milking and each scorpion venom extraction were performed 5 times with intervals of 30–45 days. An extractor with a dimmer potentiometer was developed for this study. The scorpion was placed in the restraining device (an acrylic base with a metallic plate, and a plastic flexible band), the venom gland was firmly held with the pair of forceps, and the platinum electrode was placed against the distal somites. Electrical stimulation was applied for milking, using different electrical pulses (5 to 20 V) during about 10–15 s. Venom was pooled and immediately stored at −20°C.

### Reversed-Phase Chromatography of *R. crassicauda* Venom and Tris-Tricine-SDS-PAGE

The pooled desiccated *R. crassicauda* venom was dispersed in 0.5 mL of ultrapure water, centrifuged at 12,000 *g*, 4°C, during 10 min, for removal of insoluble mucus, resulting in the soluble crude venom (supernatant without mucus). The precipitate was resuspended twice under the same conditions and the supernatants were pooled. The protein concentration of the resulting soluble venom without mucus was estimated by NanoDrop^™^ 2000 (Thermo Scientific, United States) using the extinction coefficient of 1.0. The soluble venom (2 mg of proteins) was applied onto a C18 column (10 mm × 250 mm, 300 Å, 5 μm particles, Jupiter^®^ Phenomenex, United States) equilibrated with 0.1% (V/V) trifluoroacetic acid (TFA). The samples were eluted with a step concentration gradient from 0 to 100% of solution B (80% acetonitrile, ACN, in 0.1% TFA), at a flow rate of 5 mL/min. Absorbance was monitored at 214 nm by the FPLC Äkta Basic UPC-10 Frac-920 system (GE Healthcare, Uppsala, Sweden). The eluted fractions were lyophilized and stored at −20°C until use. The major peak eluted in this chromatographic step was rechromatographed on another C18 column (250 × 2.1 mm, 300 Å, 5 μm particles, Jupiter^®^ Phenomenex, United States), at a flow rate of 0.5 mL/min. The major isolated toxin was designated as Rc1 and submitted to next assays. Protein recovery of Rc1 was calculated by the relative peak area (the fraction peak area divided by total area of all the fractions in the chromatogram), considering both chromatograms. Soluble venoms (*T. serrulatus* venom, TsV, and RcV, 18 μg/well), Ts1 (2 μg/well), and the eluted chromatographic fractions (2 μg/well) were analyzed under reducing conditions by Tris Tricine Sodium Dodecyl Sulfate Polyacrylamide Gel Electrophoresis (Tris-Tricine-SDS-PAGE, 16.5%) (74). The gels were stained with PlusOne Coomassie Blue PhastGel^®^ R-350 (GE Healthcare, Uppsala, Sweden).

### Mass Spectrometry Analysis

The molecular mass of Rc1 (0.65 μg) was determined by MALDI with TOF analyzer (RapifleX, Bruker Corporation, Billerica, MA, United States) controlled by flexControl 4.0 software (Bruker Corporation, Billerica, MA, United States). The parameters to obtain data were 10,000 laser shots per spectrum, 500 Hz laser frequency, and the instrument operating in linear positive mode. RapifleX was calibrated with Protein Calibration Standard I (∼4000 and 20000 Da, Bruker Corporation, Billerica, MA, United States). As matrix, 10 mg/mL solution of 2,5-dihydroxybenzoic acid (DHB) was prepared in ACN and 0.1% TFA at 1:1 ratio. Data analysis was performed through the software flexAnalysis 3.4 (Bruker Corporation, Billerica, MA, United States).

Rc1 (1.5**μ**g) was also reduced, alkylated, and digested with trypsin (Thermo Fisher Scientific Inc., Waltham, MA, United States) at 1:50 ratio, overnight, under 600 rpm at 37°C. Additionally, a second digestion was performed under similar conditions, but with a different ratio and time (1:100 and 3 h, respectively). The reaction was stopped with 0.5% TFA and the sample desalted. Desalted tryptic peptides were solubilized in 50% ACN and 0.1% TFA solution and analyzed by nLC-MS/MS in a Acquity UPLC^®^ M-Class (Waters, Milford, MA, United States) coupled with a Q-Exactive^™^ Plus Hybrid Quadrupole-Orbitrap^™^ Mass spectrometer (Thermo Scientific, Bremen, Germany). They were eluted at a flow rate of 0.6 nl/min using an ACN gradient (3–80%) in 0.1% formic acid for 130 min and immediately submitted to mass spectrometry analysis. MS spectra (400–1750 *m/z*) were acquired with high resolution (70,000 at *m/z* 200) and automatic gain control (AGC) target of 3e6. The twelve most intense ions were subsequently fragmented by HCD in a data-dependent mode. MS/MS spectra (200–2000 *m/z*) were acquired with resolution of 17,500 (at *m/z* 200), normalized collision energy of 25, AGC target of 1e5 and isolation window of ±2 *m/z*. Ions with not assigned or **+**1 charge were not fragmented. Data were analyzed by PEAKS Studio 7 software (Bioinformatics Solutions Inc., Waterloo, Canada) and peptides sequences were generated by automatic *de novo* sequencing setting the following parameters: parent and fragment mass error tolerance (5.0 ppm and 0.015 Da, respectively) and fixed (cysteine carbamidomethylation) and variable (deamidation of Asn and Gln and oxidation of Met) modifications. All results were manually confirmed to exclude false positives and spectra were also manually investigated.

### N-Terminal Sequencing

The N-terminal sequence of Rc1 (33 μg) was determined by Edman degradation (76), using an automated protein sequenator model PPSQ-33A (Shimadzu Co., Kyoto, Japan). The obtained sequence was compared with databases, searching similarities by using Basic Local Alignment Search Tool (BLAST)^1^.

### Hyaluronidase Activity

The soluble venoms of *T. serrulatus* and *R. crassicauda* (5 μg/well) were analyzed under non-reducing conditions on Tris-Tricine-SDS-PAGE gel (74) and stained with PlusOne Coomassie Blue PhastGel^®^ R-350. To detect the presence of hyaluronidase, a 16.5% separating gel containing 0.4 mg/mL hyaluronan, overlaid by a 5% stacking gel was used (67), which was stained with Stains-all (Sigma Chemical Co., St. Louis, United States) for evaluation of the hyaluronidase activity (76).

### Phosphodiesterase Activity

Phosphodiesterase activity in *R. crassicauda* venom (5 μg) was determined in a 96-well plate by using *bis*(p-nitrophenyl) phosphate as substrate, according to the protocol described by Björk (77) and modified by Valério et al. (78), with absorbance reading at 400 nm. As a positive control, snake venom PDE (0.75 μg) was used.

### Phospholipase A_2_ Activity

The phospholipase A_2_ (PLA_2_) activity was evaluated according to Habermann and Hardt (79). Briefly, one part of fresh egg yolk was mixed with 3 parts (V/V) of phosphate buffered saline (PBS) and centrifuged at 2,000 *g* for 10 min. Then, 1.25 mL of the supernatant was added to a final 25 mL-suspension containing 1.5% agar and 0.25 mM CaCl_2_ in PBS and poured into plastic Petri dishes (90 × 15 mm, flat bottom). After layer consolidation, cylindrical holes were performed using a 10 μL-pipet tip. Each well was charged with 50 μL of *R. crassicauda* venom (65 μg) and as controls bovine serum albumin (300 μg) and *Crotalus durissus terrificus* venom (10 μg) were used. The plates were incubated at 37°C for 16 h and the halo diameter corresponding to the phospholipase activity was measured.

### Antivenom Cross-Reactivity

ELISA 96-well plate (Costar, Corning, New York, United States) was coated with *T. serrulatus* venom, *R. crassicauda* venom, or *R. crassicauda* chromatographic fractions (2 μg/well) in 0.05 M carbonate/bicarbonate buffer, pH 9.6 (100 μL/well), and incubated overnight at 4°C. Control wells were coated with non-immune horse serum (diluted 1:50 in 0.05 M carbonate/bicarbonate buffer, pH 9.6, 100 μL/well), scorpion antivenom, or arachnid antivenom, which also includes antibodies specific to *T. serrulatus* scorpion venom; Butantan Institute, SP, Brazil). The plates were washed 3 times with PBS pH 7.2, blocked by adding 250 μL of PBS containing 2% (w/V) non-fat dry milk (Molico, Nestlé, Bebey, Switzerland – MPBS), and incubated for 2 h at 37°C. Plates were washed 3 times with PBS-0.05% Tween (PBS-T) and 3 times with PBS. Then, scorpion or arachnid antivenoms (diluted 1:1000 in 1% MPBS) were added, following 1 h incubation at 37°C. The plates were washed as previously and 100 μL of anti-horse polyclonal antibodies conjugated with peroxidase (IgG-HRP, A6917, Sigma-Aldrich, St. Louis, MO, United States, 1:3000 in 1% MPBS) were added to the wells following 1 h incubation at room temperature. The plates were washed again with PBS-T and PBS. In each well, 100 μL of OPD-H_2_O_2_ (SIGMAFAST^™^ OPD tablet, Sigma-Aldrich, St. Louis, MO, United States) were added and incubated for 15 min at room temperature for color development. The reaction was stopped with 50 μL of 1 M H_2_SO_4_ (Merck, Kenilworth, NJ, United States) and absorbance was measured at 490 nm. The assay was carried out in duplicate and the results were analyzed by GraphPad Prism 8.4 software (La Jolla, CA, United States), using one-way ANOVA, followed by Tukey’s *post hoc* test.

### Cell Line and Culture

Mice macrophages J774.1 cell line (ATCC, Rockville, MD, United States) were cultured in RPMI-1640 medium supplemented with 10% (V/V) fetal bovine serum (FBS), and 1% (w/V) gentamicin, under standard conditions (37°C, 5% CO_2_, and 95% humidity). Approximately 2.5 × 10^4^ cells diluted in 100 μL of medium were plated per well and incubated overnight under the standard conditions. The medium was aspirated and replaced by medium without FBS (100 μl per well) containing *T. serrulatus* venom (TsV), Ts1, *R. crassicauda* venom (RcV), or Rc1 (100 μg/mL), and incubated for 24 h at standard conditions.

### Cytotoxicity and Cytokine Levels

The viability of J774.1 cells was determined using the 3-(4,5-dimethylthiazol-2-yl)-2,5-diphenyltetrazolium bromide (MTT) colorimetric assay (80). After 24 h of incubation with different stimuli (see cell line culture Section), 5% MTT in RPMI was added to the plated cells. Following 3 h of incubation with MTT, 50 μL of 20% sodium dodecyl sulphate (SDS) in 0.01 M HCl were added and cells were kept at room temperature until complete precipitate solubilization. Absorbance was measured at 570 nm and viability was expressed as the percentage (%) compared to unstimulated cells.

Concentrations of TNF-α, IL-6, and IL-1β were evaluated from the cell supernatants by ELISA using specific antibodies (purified and biotinylated) and cytokine standards, according to the manufacturers’ instructions (R&D Systems, MSP, United States).

### Protein Expression by Western Blotting

After removing the supernatants, J774.1 cells were lysed in radioimmunoprecipitation assay buffer RIPA buffer (Merk, Darmstadt, Germany) containing protease and phosphatase inhibitors. Protein quantification was performed using detergent-compatible methodology (DC Protein Assay, Bio-rad, CA, United States). Proteins were separated by polyacrylamide gel electrophoresis (Bolt Bis-Tris 4–12% Plus Gel, Life Technologies, CA, United States) and transferred to 0.22 μm nitrocellulose membrane (GE Healthcare, Madison, WI, United States). The membranes were blocked in Tris buffered saline (TBS) solution containing 0.01% Tween, and 5% non-fat dry milk (Molico, Nestlé, Bebey, Switzerland). Recombinant anti-NF-κB p65 antibody [E379] (Abcam, United States) and Phospho-NF-κB p65 (S536; Abcam, United States) were added at 1:5000 dilution in blocking solution; Anti-GAPDH clone (71.1; Sigma-Aldrich, St. Louis, MO, United States) was added at 1:20,000 dilution. HRP-conjugated antibodies (KPL, Gaithersburg, MD, United States) were used at the dilution of 1:5000. ECL (GE Healthcare, Chicago, IL, United States) was used for band detection. Quantification was performed using Software ImageJ 1.52a (NIH, MD, United States). Data are representative of arbitrary units relative to the control (GAPDH).

### Expression of Voltage-Gated Ion Channels in *Xenopus laevis* Oocytes

For the expression of Nav channels (hNav1.1, rNav1.2, rNav1.4, hNav1.5, mNav1.6, the invertebrate channel BgNaV1.1 and the auxiliary subunits rβ1, hβ1, and TipE) in *X. laevis* oocytes, the linearized plasmids were transcribed by using the T7 or SP6 mMessage-mMachine Transcription Kit (Thermo Fisher Scientific, United States). The harvesting of stage V–VI oocytes from anesthetized female *X. laevis* frogs was as previously described (81). Oocytes were injected with 50 nL of cRNA at a concentration of 1 ng/nL by using a microinjector (Drummond Scientific Company, Broomall, PA, United States). The oocytes were incubated in a solution containing (in mM): NaCl, 96; KCl, 2; CaCl2, 1.8; MgCl2, 2; and HEPES, 5 (pH 7.4). This solution was supplemented with 50 mg/L gentamicin sulfate. The use of *X. laevis* was approved by the Ethical Committee for animal experiments of the University of Leuven (P186/2019).

### Electrophysiological Assays

Sodium currents were recorded using the two-microelectrode voltage-clamp technique (TEVC) at room temperature (20–25°C). The recordings were processed by a GeneClamp 500 amplifier (Axon Instruments, United States) controlled by a pClamp data acquisition system (Axon Instruments, United States). Whole-cell currents from oocytes were recorded 1–5 days after injection. Currents and voltage electrodes had resistances from 0.8 to 1.4 MΩ and were filled with 3 M KCl. Currents were sampled at 20 kHz and filtered at 2 kHz using a four-pole low-pass Bessel filter. Leak subtraction was performed using a −P/4 protocol. For the assays, Rc1 diluted in ND-96 solution was added directly to the recording chamber to obtain the desired final concentration (1 μM). Experiments were performed at least three times.

For the activation protocols, 100 ms test depolarization, ranging from −90 mV to +70 mV, were applied from a holding potential of −90 mV, in 5 mV increments at 5 s intervals. For the inactivation protocols, double pulses with a conditioning pulse applied from a holding potential of −100 mV to a range of potentials from −90 mV to 0 mV, in 5 mV increments for 50 ms, immediately followed by a test pulse to 0 mV (or −5 mV) were employed. Data were normalized to the maximal Nav current amplitude (Imax), plotted against the pre-pulse potential and fitted using the Boltzmann equation: I_Na_/I_max_ = {(1-C)/(1 + exp[(V-V_h_)/k)]} + C, where I_max_ is the maximal I_Na_, V_h_ is the voltage corresponding to half-maximal inactivation, V is the test voltage, k is the slope factor, and C is a constant representing a non-inactivating persistent fraction (close to 0 in control).

### Nociceptive Assays

Injections of 0.01 mL of *R. crassicauda* venom (1, 3, and 9 μg) and Rc1 (0.5, 2, and 4 μg) into the plantar surface (ipl) of the right hind paw of C57BL/6 mice (male, 18–22g, *n* = 5) were performed. Control groups received ipl injections of TsV (1 μg), Ts1 (0.5 μg), or physiological solution (0.9% NaCl). The time mice spent either licking or lifting/shaking the injected paw was recorded at 5 min intervals for 35 min. All experiments were conducted according to the guidelines of the Ethic Principles in Animal Experimentation of School of Medicine of Ribeirão Preto – University of São Paulo, with the license number 246/2019.

### Statistical Analyzes

The experiments were performed at least in triplicate and the results were expressed by standard deviations (SD). The statistical significance of the results was assessed using analysis of variance (one-way ANOVA or two-way ANOVA) followed by Tukey *post hoc* test through the GraphPad Prism 8.4.3 software. *P* values < 0.05 were considered significant.

## Data Availability Statement

The datasets presented in this study can be found in online repositories. The names of the repository/repositories and accession number(s) can be found in the article/ Supplementary Material.

## Ethics Statement

The animal study was reviewed and approved by Comissão de Ética no Uso de Animais (CEUA-FMRP).

## Author Contributions

MP, KB, JT, TC, and EA designed the research. CA, KB, FC, IO, CB, GA-S, KZ, MR, EP-J, and SP performed the research. MP, KB, IO, GW, SP, LQ, LF, and UZ analyzed the data. All authors contributed to writing of the manuscript.

## Conflict of Interest

The authors declare that the research was conducted in the absence of any commercial or financial relationships that could be construed as a potential conflict of interest.

## Footnotes

1 http://blast.ncbi.nlm.nih.gov/Blast.cgi
