# Supplementary material for: Pioneering Study on Rhopalurus crassicauda Scorpion Venom: Isolation and Characterization of the Major Toxin and Hyaluronidase
Source: Front Immunol. 2020 Aug 20;11:2011. doi: 10.3389/fimmu.2020.02011 (PMC7468477; doi:10.3389/fimmu.2020.02011)
Supplement: Supplementary file 1 [file Table_1.docx]

**Supplementary Table 1.** Rc1 internal peptides obtained by MS/MS and *de novo* sequencing. Leucine and isoleucine residues cannot be differentiated by the mass spectrometer used. So, the assignment was made with the N-terminal sequence through Edman degradation for those peptides or homology matching. Carbamidomethylated cysteines are represented by C. Deamidated asparagine residues (represented by N) was determined based on the N-terminal sequence by Edman degradation and homology matching.

| Peptide | Spectral counting | Experimental molecular mass | Theoretical molecular mass | *z* | Mass variation (ppm) |
| --- | --- | --- | --- | --- | --- |
| GCKISCVINNEYCSR | 1 | 930.4148 | 930.4135 | +2 | 1.3972 |
| KISCVINNEYCSR | 1 | 822.3789 | 822.3794 | +2 | -0.6080 |
| KISCVINNEYCSR | 1 | 821.8878 | 821.8874 | +2 | 0.4867 |
| ISCVINNEYCSR | 41  2 | 757.8396  505.5630 | 757.8400  505.5624 | +2  +3 | -0.5278  1.1868 |
| ISCVINNEYCSR | 3 | 758.3342 | 758.3320 | +2 | 2.9011 |
| ISCVINNEYCSR | 1 | 758.8276 | 758.8240 | +2 | 4.7442 |
| ISCVINNEYCSR | 4 | 758.3351 | 758.3320 | +2 | 4.0879 |
| SCVINNEYCSR | 1 | 701.2982 | 701.2979 | +2 | 0.4278 |
| CVINNEYCSR | 1 | 657.7819 | 657.7819 | +2 | 0 |
| SGYCYFLR | 5 | 533.2455 | 533.2448 | +2 | 1.3127 |
| WGLACWCDGVPPQR | 2 | 851.3854 | 851.3849 | +2 | 0.5873 |
| ACWCDGVPPQR | 2 | 673.2929 | 673.2924 | +2 | 0.7426 |
| WCDGVPPQR | 11 | 557.7592 | 557.7586 | +2 | 1.0757 |
